# High anorectal malformation in a five-month-old boy: a case report

**DOI:** 10.1186/1752-1947-4-296

**Published:** 2010-08-31

**Authors:** Anand Pandey, Ajay N Gangopadhyay, Vijayendra Kumar, Shiv P Sharma

**Affiliations:** 1 Department of Pediatric Surgery, Institute of Medical Sciences, Banaras Hindu University, Varanasi, 221005, UP, India

## Abstract

**Introduction:**

Anorectal malformation, one of the most common congenital defects, may present with a wide spectrum of defects. Almost all male patients present within first few days of life.

**Case presentation:**

A five-month-old baby boy of Indian origin and nationality presented with anal atresia and associated rectourethral prostatic fistula. The anatomy of the malformation and our patient's good condition permitted a primary definitive repair of the anomaly. A brief review of the relevant literature is included.

**Conclusion:**

Delayed presentation of a patient with high anorectal malformation is rare. The appropriate treatment can be rewarding.

## Introduction

Anorectal malformation (ARM) is one of the most common congenital defects having an incidence of between one per 1500 and one per 5000 live births [1,2]. This anomaly is characterized by an absent anal opening: the rectum may either communicate with the urinary tract by a fistula or end blind.

ARM may present with a wide spectrum of defects, ranging from relatively low malformations to very complex high defects [2]. ARMs are usually diagnosed at birth. If not, almost all male patients present within the first few days of life with obstructive symptoms because of absent or narrow fistula. We present a male patient of high ARM, who exceptionally presented at the age of five months.

## Case presentation

**Figure 1:**
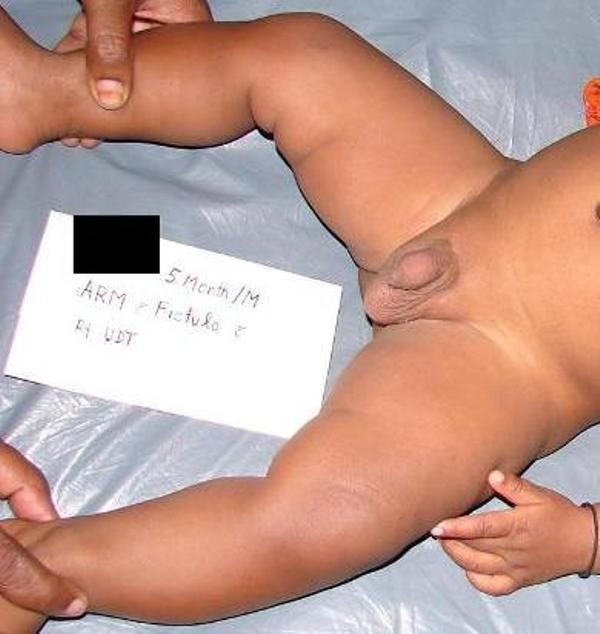
**Patient of high ARM**. The patient appears to be healthy. His abdomen is soft.

**Figure 2:**
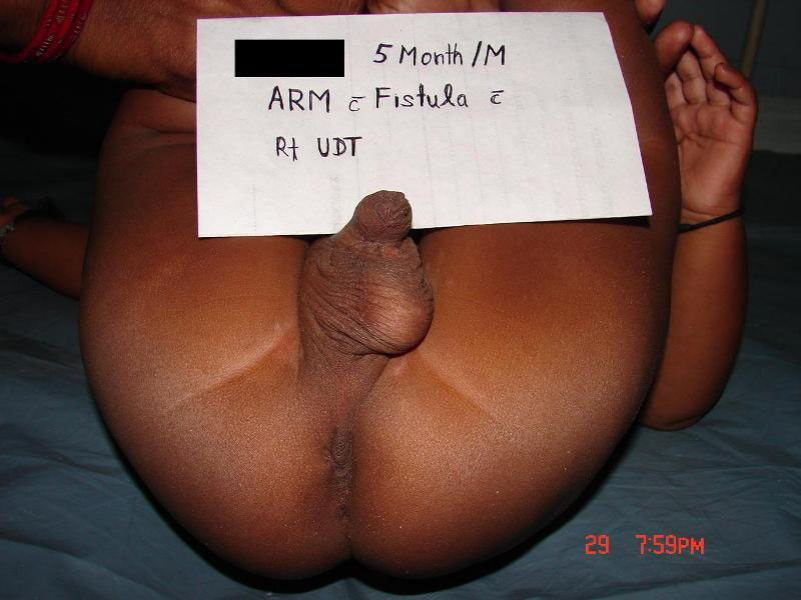
**Perineal view of the patient**. Absent anal opening and right undescended testis are obvious.

A five-month-old baby boy of Indian origin and nationality presented to the department of Pediatric Surgery at the Institute of Medical Sciences, Banaras Hindu University with absent anal opening, along with passage of flatus and feces through the urethra since birth without any problem (Figures 1 and 2). On examination, his abdomen was soft and the right undescended testis was palpated in the inguinal canal. He was also passing clear urine intermittently. No other anomalies were noticed. Abdominal ultrasound showed normal findings. The babygram revealed no bony abnormality. Bowel gas was seen up to the pelvis.

**Figure 3:**
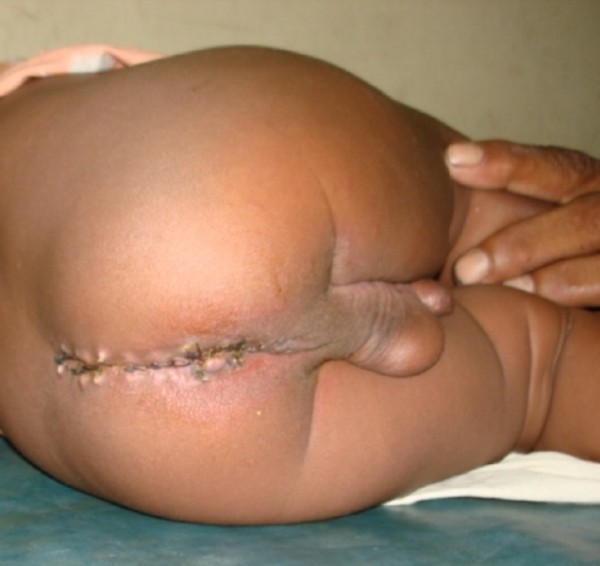
**Post-operative view of the patient**. Suture line is healthy.

Our patient was planned for the primary posterosagittal anorectoplasty (PSARP). Intra-operatively, a large rectourethral prostatic fistula was found and closed. The post-operative period was uneventful (Figure 3) and the patient was discharged in a satisfactory condition.

The bad financial conditions of the family led to the delayed presentation of the child to a specialist.

## Discussion

The most unusual fact of this case is the age of presentation. As mentioned previously, male patients with ARM usually present within the first four or five days of life. This is due to the presence of a narrow fistulous communication between the rectum and the urinary tract, which does not allow the bowel to decompress.

Although the diagnosis of ARM is made at birth [3], the situation is different in our country, where a large number of deliveries take place at home. This may cause a delay in diagnosis as perineal examination is not a routine in the countryside. The delay in diagnosing this condition may lead to death [4,5].

A (long-term) study noticed [3] that all patients with delayed diagnosis had low type ARM. However, this was not the case in our patient. The absence of the symptoms despite high type of ARM is attributed to the wide fistulous communication between the rectum and the urinary tract. It can be argued that primary surgery is not indicated in a patient with a rectourethral prostatic fistula having delayed presentation because of possible proximal bowel dilation. The soft and non-distended abdomen (Figure 1) led us to the pre-operative presumption of non-dilated rectum. The presumption was confirmed intra-operatively and explained by the existence of a large fistula leading to the facile decompression of the bowel. The abdominal approach was not attempted as our patient was also passing clear urine, which can not be the case if there was a colovesical fistula. The successful operation also confirms our view.

This case has a few peculiarities. First, it is probably the oldest ever reported case of high ARM presenting at five months of age; the previous case presented at 45 days of life [6]. Second, primary PSARP is feasible even at this age, though we agree that experience is needed in dealing with it; our centre has the necessary expertise to carry out primary PSARP [7]. This case also highlights the impact of financial condition, which can hinder a patient to seek treatment.

## Conclusions

This is an extremely uncommon case of a patient with high ARM in respect of the anatomy of malformation and the time of presentation. A primary repair without colostomy is unusual in such patients, but proved to be appropriate and successful in this child having a large fistula and a non-distended rectum.

## Competing interests

The authors declare that they have no competing interests.

## Authors' contributions

ANG, SPS and AP operated upon the patient. AP, ANG and VK carried out the literature review. All authors read and approved the final manuscript.

## Consent

Written informed consent was obtained from the parents of the patient for publication of this case report and any accompanying images. A copy of the written consent is available for review by the journal's Editor-in-Chief.
